# CRUMB: a shiny-based app to analyze rhythmic feeding in *Drosophila* using the FLIC system

**DOI:** 10.12688/f1000research.132587.1

**Published:** 2023-04-06

**Authors:** Sergio Hidalgo, Joanna C Chiu

**Affiliations:** 1 Entomology and Nematology, University of California Davis, Davis, California, 94534, USA

**Keywords:** Feeding-fasting rhythm, FLIC, Shiny, Drosophila, Circadian clock, chrononutrition, time-restricted eating

## Abstract

Rhythmic feeding activity has become an important research area for circadian biologists as it is now clear that metabolic input is critical for regulating circadian rhythms, and chrononutrition has been shown to promote health span. In contrast to locomotor activity rhythm, studies conducting high throughput analysis of *Drosophila* rhythmic food intake have been limited and few monitoring system options are available. One monitoring system, the Fly Liquid-Food Interaction Counter (FLIC) has become popular, but there is a lack of efficient analysis toolkits to facilitate scalability and ensure reproducibility by using unified parameters for data analysis. Here, we developed Circadian Rhythm Using Mealtime Behavior (CRUMB), a user-friendly Shiny app to analyze data collected using the FLIC system. CRUMB leverages the ‘plotly’ and ‘DT’ packages to enable interactive raw data review as well as the generation of easily manipulable graphs and data tables. We used the main features of the FLIC master code provided with the system to retrieve feeding events and provide a simplified pipeline to conduct circadian analysis. We also replaced the use of base functions in time-consuming processes such as ‘rle’ and ‘read.csv’ with faster versions available from other packages to optimize computing time. We expect CRUMB to facilitate analysis of feeding-fasting rhythm as a robust output of the circadian clock.

## Introduction

The fly *Drosophila melanogaster* has been used for several decades to understand the molecular and neuronal basis of behavior. ^1^^,^^2^ This animal has been instrumental in the description of the circadian clock, ^3^^–^^10^ the machinery that allows animals to maintain daily rhythms in physiology and behavior. A common behavioral output used to assess circadian rhythmicity is locomotor activity, which is considered by many as a gold standard. Commercial options such as the Trikinetics *Drosophila* Activity Monitoring System (DAMS) have made it possible to conduct high throughput screening of clock mutants ^11^^–^^13^ and functional experiments to dissect molecular and cellular mechanisms regulating circadian rhythms. ^14^^,^^15^ Nonetheless, this behavior is only one example of circadian clock behavioral output. Feeding-fasting rhythm is also strongly regulated by the clock, although mechanisms required for maintaining food intake rhythm differ from those used in the regulation of circadian locomotion. ^16^^–^^20^

In contrast to locomotor activity rhythms, technical advances to assay feeding rhythms have lagged. A popular option is the Capillary Feeder (CAFE) assay, which allows for the quantification of the amount of feeding by means of measuring the movement of a meniscus along a capillary tube. ^21^ This assay has proven useful in capturing the feeding rhythms of groups of flies; however, the distance of the meniscus is only evident when a significant amount of liquid food has been consumed and thus its temporal resolution is limited. In 2014, Scott Pletcher’s lab developed FLIC (Fly Liquid-Food Interaction Counter), a new system to assess feeding which allows for continuous monitoring (millisecond scale) and scalability, promising high throughput recordings. ^22^ Since then, several other groups have adopted the system and used it to address different biological questions including rhythmic feeding and metabolic homeostasis. ^18^^,^^23^^–^^32^

Available options of software to analyze locomotor activity from DAMS have multiplied over the years, offering different user interphases and developed in different coding languages ^33^^–^^37^ (summarized in Cai *et al.* ^15^). In contrast, due to its recent implementation, user-friendly tools to analyze FLIC data are still scarce, ^23^ and available scripts require the user to understand and use R coding syntax. Here we present CRUMB (Circadian Rhythm Using Mealtime Behavior), a shiny app based on the popular DAMS Shiny app Shiny-R DAM combined with the core FLIC code master scripts provided with the equipment ( GitHub FLIC R code). CRUMB leverages features from several packages including ‘ plotly’ and ‘ DT’, to generate easily manipulable graphs and data tables. CRUMB also uses some of the versatile tools offered by the ‘ zeitgebr’ package ^35^ to enable easy and comprehensive circadian analysis—all of this wrapped in a simple Graphical user interface (GUI) provided by the ‘shiny’ package.

## Methods

### Implementation

Initialization

Initialization of the app must be conducted from the ‘app.R’ script. On the first usage, if ‘shiny’ or any other package is not installed, running this script will prompt a message indicating the list of missing packages and asking for installation. Following this process, CRUMB will be started and ready to run. The tabs on this app are organized in the required order of use, meaning that processes in the tab ‘File loading & QC’ are required for the subsequent tab ‘Initial Survey’, which then is required for subsequent tabs and so forth.

Input files

The current version of CRUMB supports the original, unedited CSV files from the FLIC-MCU-2 system (Sable Systems International, NV). Further testing is needed to determine its suitability for other versions of the FLIC-MCU system. Given that the time resolution provided by FLIC monitors is in the scale of milliseconds, file sizes are correlated to the length of the recordings. For a full day of recordings, the file size is 44.4 MB. Although CRUMB is designed to analyze the data of several consecutive days, shorter recordings/files can be used as input but some functionalities ( *i.e.*, circadian analysis) will not be available.

**Figure 1.**
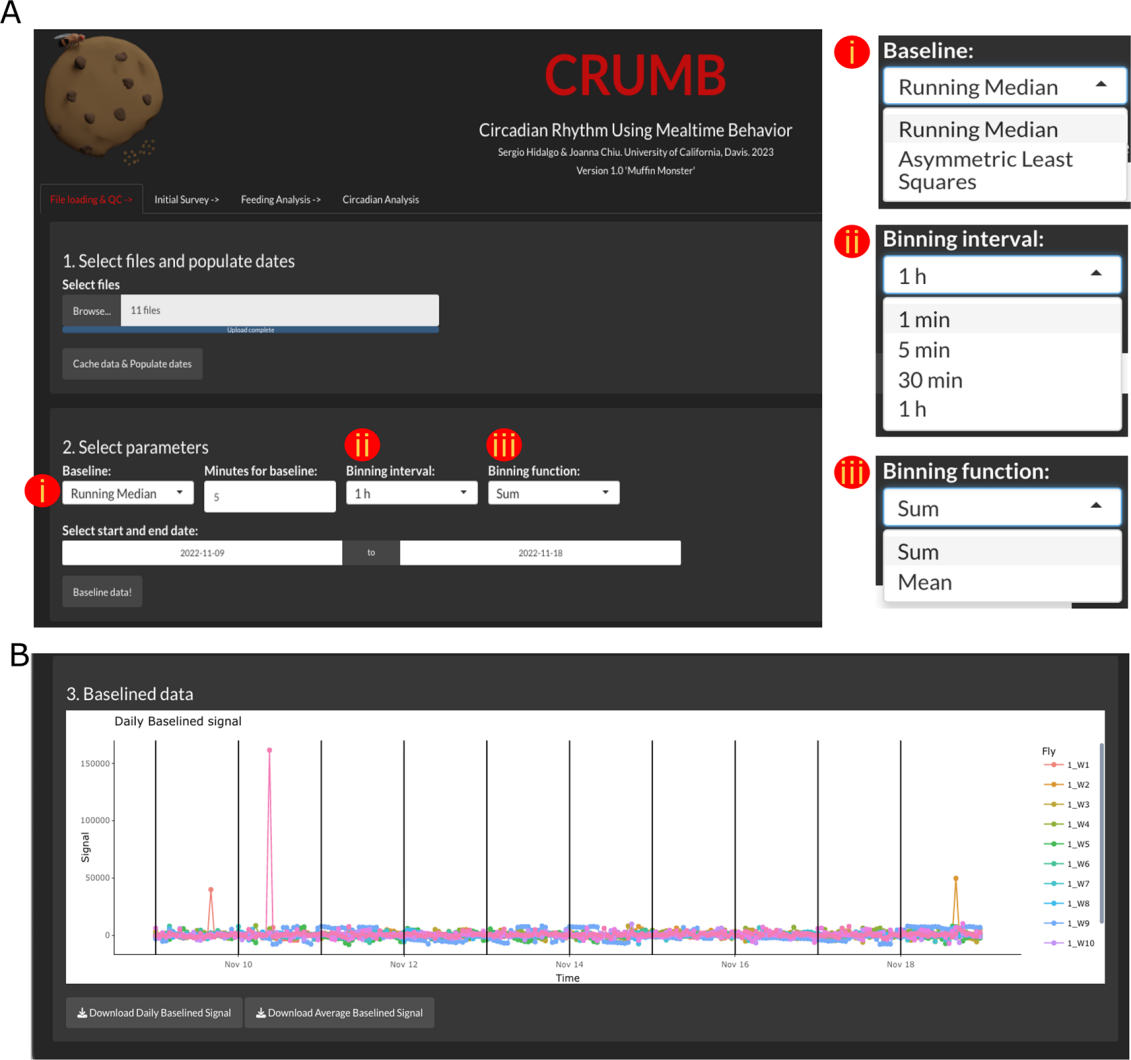
Loading and visualization of pre-processed data. (A) Raw data files and dates of the experiment are loaded in step 1. In step 2, the user can select parameters to baseline the raw data by choosing (i) a baseline function and time duration window for the Running Median option. Additional parameters including (ii) a binning interval and (iii) a binning function can be indicated for visualization purposes. (B) Baselined data are visualized using ‘plotly’ interactive graphs, allowing individual inspection of data. Data can be downloaded as CSV files.

To load the files, use the ‘Browse’ button. Select the file or files, from one or several different monitors. After loading, use ‘Cache data & Populate dates’ to engage the files and populate the dates of the experiments and monitor numbers ( Figure 1A).

Baseline data & QC

Feeding events are calculated from the baselined data obtained from FLIC monitors. In CRUMB, this baseline can be calculated using one of two functions: running median or asymmetric least squares. The running median is the original method used in the FLIC master code, adopted by CRUMB, and requires a window in minutes to calculate the median for subtraction. If desired, data can be trimmed by choosing the start and end date. This trimming is made to the raw data, preventing unnecessary processing, and therefore reducing computation time. For visualization purposes, a binning interval and a binning function are needed. By default, these values are set to ‘1 hour’ and ‘Sum’, respectively ( Figure 1A). Note that if recordings are shorter than this interval, the data will be represented as a unique point. Once these parameters are set, press ‘Baseline data!’. Graphs are compiled using the ‘ plotly’ package, an interactive interface that permits easy inspection of the graphs, allowing the selection of individual points and areas within the plots ( Figure 1B). The isolation of tracks in ‘plotly’ graphs can thus be used to visualize individual flies to look for anomalies or drifting in the baseline due to liquid food overfill.

Initial survey

**Figure 2.**
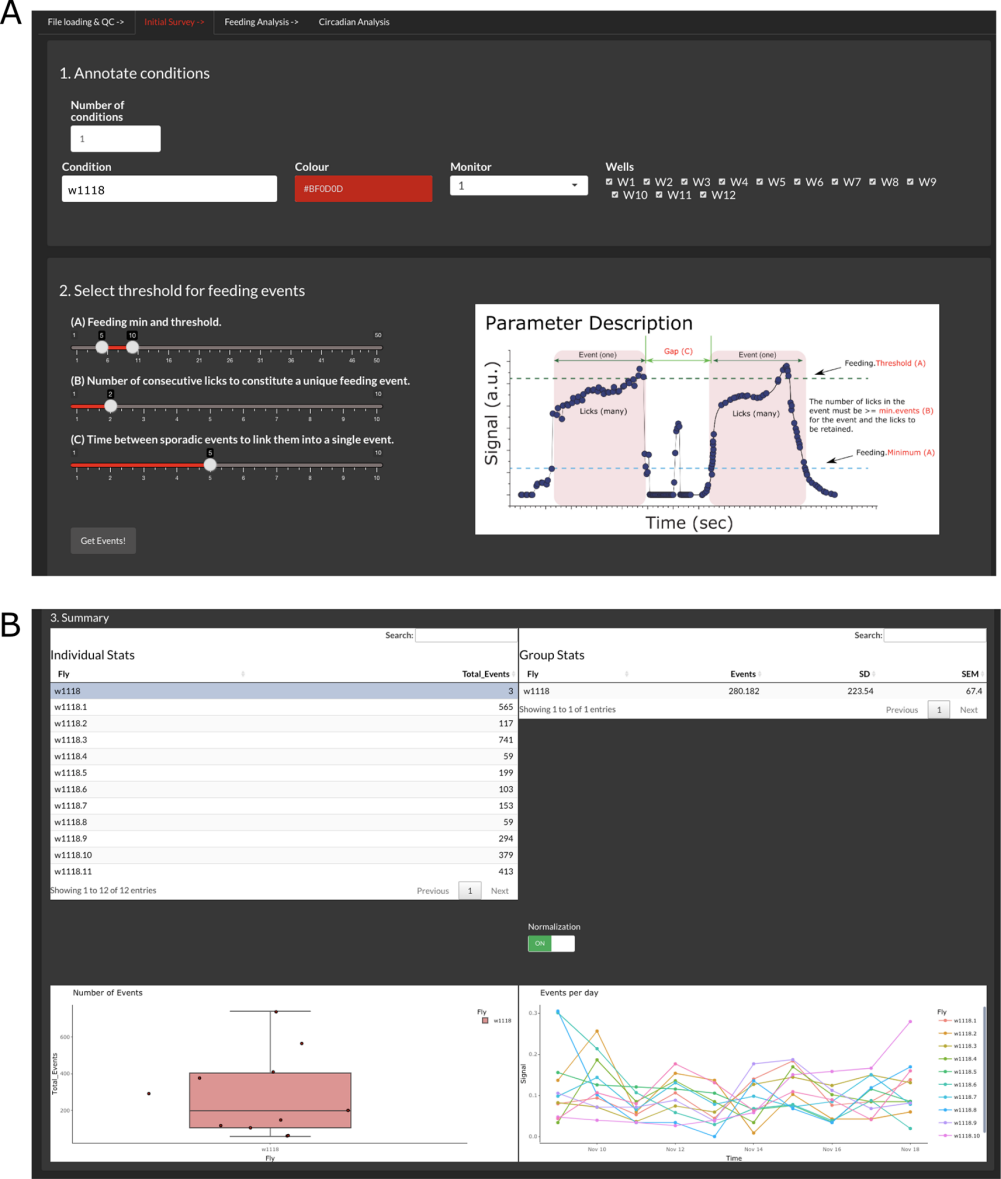
Calculating total feeding events for each condition. (A) In step 1, users can define conditions per monitor and/or wells. Step 2 allows the selection of the parameters required to calculate feeding events based on the baseline data. The ‘Parameter Description’ image (modified from FLIC code master) provides definitions for these factors. (B) After the calculation, the summary section displays the processed data as individual events per fly/well (top left), average events per group (top right and bottom left), and the number of events across days for each fly (bottom right). The feeding activity of each well can be scaled to the highest value of that well throughout the duration of the experiment by using the normalization switch. This selection is inheritable, meaning that the rest of the analysis will be conducted with the normalized data. Additionally, users can select outliers from the ‘Individual Stats’ table to exclude them from the rest of the analysis.

The tab in Figure 2A (‘Initial Survey’) allows assigning names and colors to the different conditions present in the experiment. Given that CRUMB allows input from several FLIC monitors, an option to choose the monitor is available. This is populated with the monitor number extracted from the filenames. We recommend that the users use two or more conditions per monitor to account for variability between monitors. Because of this, CRUMB allows assigning wells from the same monitor to different conditions. Use the boxes on the right to check or uncheck the wells. If the same condition is repeated in different monitors ( *i.e.*, flies from the same genotype in two or three monitors), adding another condition with the same name will allow the pooling of the data in subsequent analyses ( Figure 2A). If no names are assigned, fields will be populated automatically with the default name “Condition”.

The FLIC system is designed so events that meet certain criteria can be considered feeding events ( Figure 2A). These parameters correspond to the feeding minimum (minimum value of the baselined signal to consider an interaction), feeding threshold (threshold value that consecutive interactions need to pass to consider the event as feeding), number of consecutive licks (minimum number of interactions required to define a feeding event) and gap (time between events to consider consecutive events as one single event). The setting of these parameters can be tuned and tailored to each monitor and need to be determined empirically.

Once parameters are defined, clicking ‘Get Events!’ will initiate the calculations. This process is the most machine-demanding of the entire analysis, and the time spent on it correlates with the length of the input files. Additionally, permissive parameters will result in more events being considered, increasing the processing time.

The output summary is composed of four main elements that allow the inspection of the data ( Figure 2B). First, an ‘Individual Stats’ table and a ‘Group Stats’ table are displayed to show the total number of events per fly and the average, standard deviation (SD), and standard error of the mean (SEM) for the groups indicated in the previous step, respectively. Second, two graphs are displayed, a boxplot of the total events per condition showing individual points and a graph of events per day throughout the duration of the experiment. Given the variability of the data, it is possible to enable or disable normalization, which divides the binned events of each fly by the sum of all the events per fly. This will impact the display of the feeding events and average event profiles in the next tab, ‘Feeding Analysis’, and the calculations in the ‘Circadian Analysis’ tab.

The function of this summary is two-fold: 1) it allows for inspection of the data for each individual fly and pooled data and 2) allows for the exclusion of dead flies. The criterion to consider dead individuals may vary but data from this section can provide guidance. For example, from the ‘Individual Stats’ table, flies that fail to show any events above a threshold defined by the experimenter can be excluded. Also, the boxplot of total events facilitates the determination of outliers as they will be marked by a black dot next to the data point. Finally, flies that do not survive for the entire experiment can be excluded. This information can be extracted from the ‘Events per day’ graph. To exclude individuals from the rest of the analyses, simply select them in the ‘Individual Stats’ table by clicking them.

Feeding analysis

**Figure 3.**
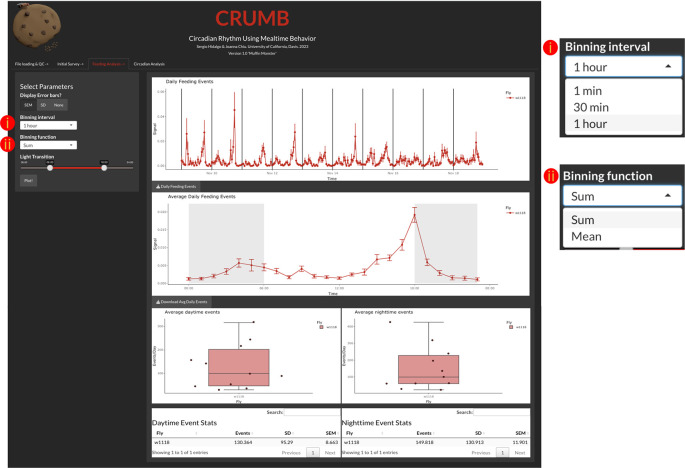
Determining feeding activity over time. Users can access and visualize the feeding events across the duration of the experiment (top graph) with the time resolution defined by (i) a binning interval and (ii) a binning function. The daily feeding events are averaged and displayed as an activity profile graph (middle graph) with gray backgrounds indicating when the lights are off. The duration of day and night can be selected under ‘Light Transition’ from the left panel where the region between the two sliders is when the light is on (red line). This selection also serves to calculate the daytime feeding events (bottom left graph and table) and nighttime feeding events (bottom right graph and table) per well. Error bars for the top two graphs can be defined by the user as Standard Error of the Mean (SEM), Standard Deviation (SD), or can be omitted if desired, by selecting ‘None’. Additionally, data are available as CSV files for download using the download buttons below each graph.

The ‘Feeding Analysis’ tab ( Figure 3) displays binned events across several days of recordings and average daily events (feeding profile). To access these, the user must indicate a binning interval (1 min, 30 min, or 1 hour), binning function (Sum or Mean), and descriptive statistic represented by the error bars (SEM, SD, or None) ( Figure 3). The generated graphs are also interactive and allow the resizing of the axis and zoom of particular windows within the graph.

The user can indicate the time of lights on and off using the light transition feature. This information is used to determine which events occurred during the day and which events occurred during the night. This information is summarized in data tables and in boxplots ( Figure 3).

Circadian analysis

**Figure 4.**
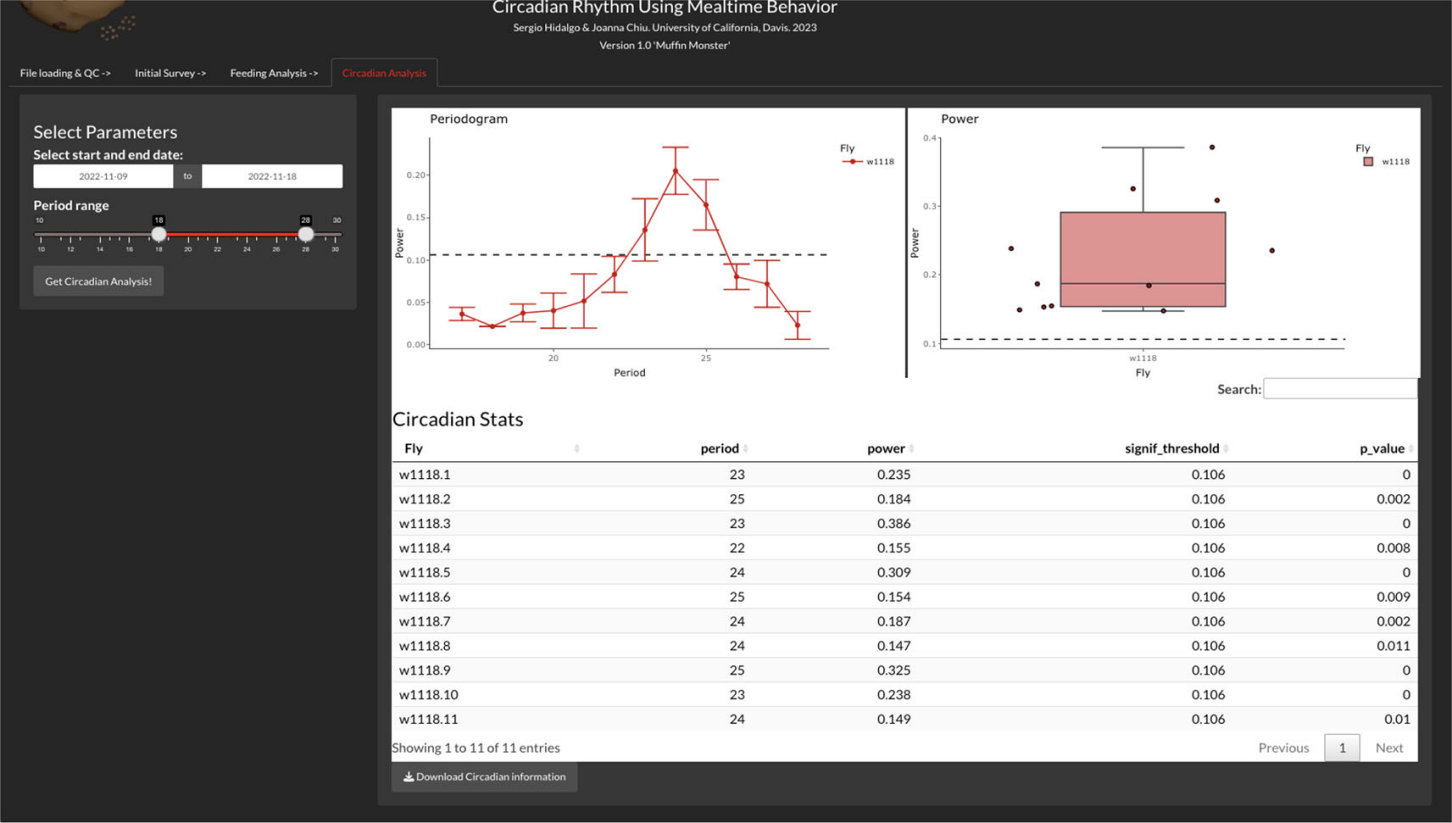
Circadian analysis of feeding activity. In this tab, users can calculate the rhythmicity of feeding events from data collected under constant darkness using autocorrelation analysis. A range of possible period lengths can be specified for this calculation. An average periodogram (top left) and a graph of the maximum power values per well (top right) are displayed. Dotted lines indicate the threshold for the significance of periods and error bars are displayed as SEM. A summary table with this information is also provided (bottom) and can be downloaded as a CSV file.

The circadian analysis is carried out in the ‘Circadian Analysis’ tab, and the current version of CRUMB allows the setting of the start and end date of the constant darkness (DD) period and a slider to choose the range of periods (in hours) that are included in the circadian analysis ( Figure 4). CRUMB uses the autocorrelation function estimation to get the period and power for rhythmicity from the ‘stats’ package wrapped in the periodogram function from the ‘ zeitgebr’ package.

The output of this analysis is a periodogram graph, showing the period over the power from the autocorrelation function and a boxplot of the highest power for each condition. Finally, a table (exportable as CSV) is obtained with the period, the highest power for each fly, and the p-value for that period estimation. The significant threshold (signif_threshold) is also shown in the table and is displayed as dotted lines in the periodogram and the power graphs ( Figure 4).

Operation (minimal system requirements needed to run the software)

CRUMB was developed with R version 4.0.3 using the FLIC master code version 4.0 from the Pletcher lab as a backbone, modified and wrapped in a shiny app to manage the GUI using Shiny version 1.7.4. No internet connection is required to run the app but if any required package is missing, the user will be prompted with the option to download and install them from CRAN, and the completion of these actions requires internet access. Output graphs can be obtained from ‘plotly’ features as PNG files.

Output tables are in CSV format and can be opened using any compatible spreadsheet application including Microsoft Excel, Apple Numbers, or any other spreadsheet application.

### Use cases

Default: determining rhythmicity

**Figure 5.**
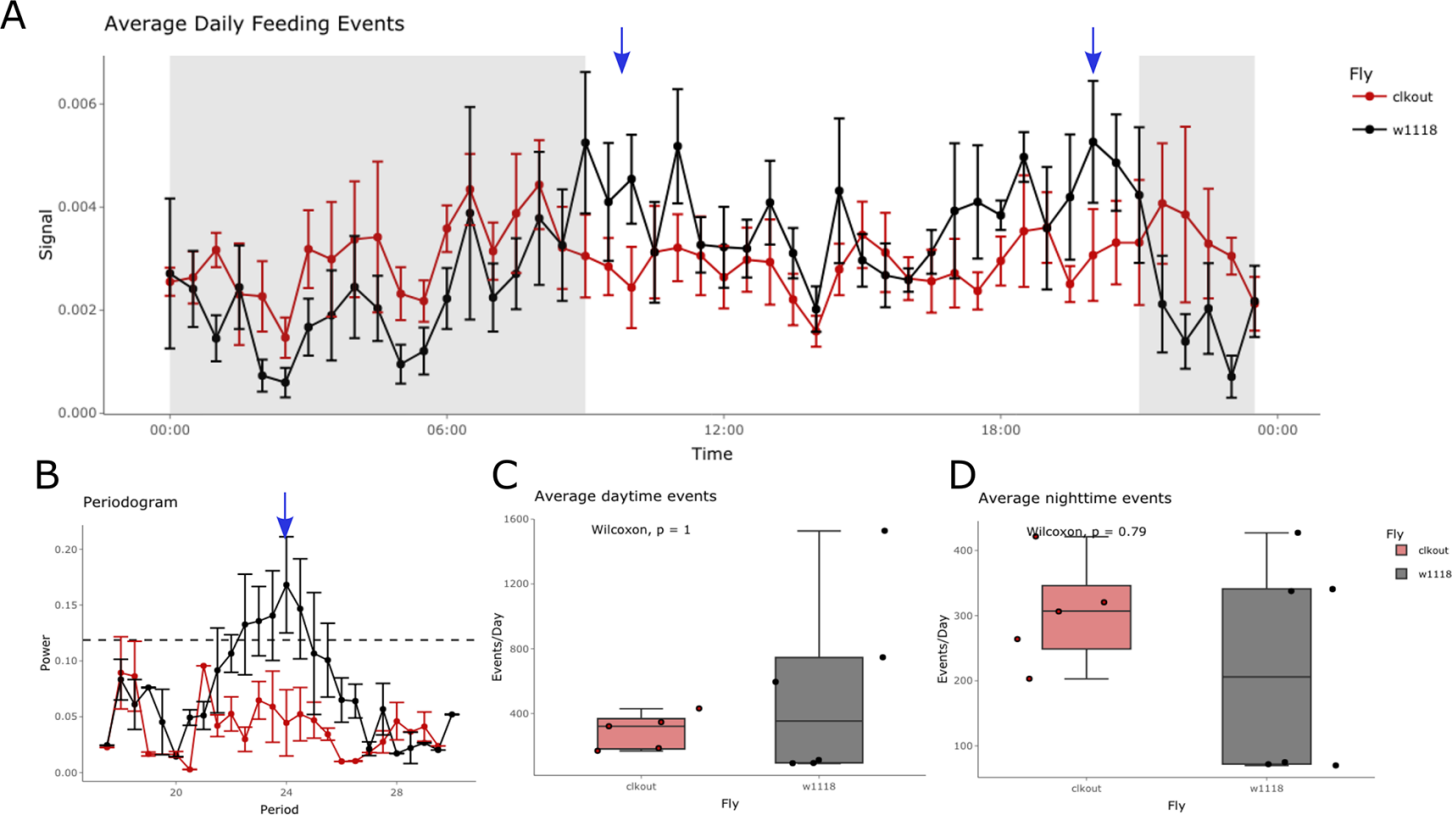
Feeding activity of an arrhythmic mutant calculated using CRUMB. (A) Feeding activity was recorded for 2 days under 12:12 light-dark cycles followed by 4 days in constant darkness (DD) for a circadian mutant ( *clk ^out^*, red) and control flies ( *w ^1118^*, black). Average activity across days for control flies shows increased feeding activity anticipating lights on (09:00, blue arrow) and lights off (21:00, blue arrow), which is absent in mutant flies. (B) Control flies show a 24 h period of feeding activity as seen in the periodogram (black lines, blue arrow), a feature that is not observed for the mutant flies (red line). The dotted line represents the threshold for significance in the autocorrelation analysis. Analysis of average daytime (C) and nighttime (D) feeding events shows no difference between mutant (red) and control (black) flies. Error bars in A and B are displayed as SEM.

CRUMB can be used to determine differences in the number of feeding events as a means to understand the motivation and innate status of flies or survey rhythmic feeding behavior to understand circadian control of feeding. Here we show one example of CRUMB used to determine feeding rhythmicity in a control strain ( *w ^1118^*) compared with mutant flies with a defective circadian clock ( *w ^1118;^ clk ^out^*; annotated as *clk ^out^*). ^38^ In this example, CSV files collected from FLIC Monitor 1 across 7 full days were used as input. Wells from 1 to 6 correspond to *clk ^out^* flies while wells 7 to 12 correspond to *w ^1118^* flies. Further, default settings for all parameters for baseline calculation and for feeding event detection was used; a threshold of 5 was set as the minimum and 15 was chosen as the top limit. Flies were kept in 12 hours light:12 hours dark (12:12 LD) cycles for three days, with lights on at 09:00 am and lights off at 09:00 pm, and then changed to constant darkness for four days. As expected, feeding events for *w ^1118^* flies peak close to the light transition ( Figure 5A), both at dawn and dusk. Although this is different from reports showing only a single peak at dawn using the CAFE assay, ^20^^,^^39^^,^^40^ this result is consistent with other articles using the FLIC assay. ^18^^,^^22^^,^^32^ This discrepancy highlights the advantage of using an assay with high time resolution such as FLIC. Consistent with previous studies, ^39^ while *w ^1118^* flies show a circa 24 period in feeding behavior ( Figure 5B; black line) *clk ^out^* mutants display arrhythmic feeding behavior ( Figure 5B; red line). This is not due to a reduction in feeding events as no differences are found in the average number of daytime events or the average number of nighttime events ( Figure 5C and D, respectively).

## Conclusions

The development of FLIC offers a new, high throughput option for continuous measurement of feeding events. ^22^ In contrast to DAMS for locomotor activity, FLIC system lacks easy-to-use options for data analysis. Here, we addressed this gap by developing CRUMB, a Shiny-based app for FLIC data analysis. Our platform offers a pipeline to analyze feeding events over several days and extract key rhythmic data such as day and night feeding events and feeding rhythmicity.

CRUMB uses the FLIC master code backbone, which offers compatibility with previous analysis. Also, we increased calculation speed by dropping some collected data, such as the number of licks or time of feeding events. We believe that this trade-off is crucial when analyzing long recordings necessary for circadian rhythm analysis, which require longer computing time. Although these parameters have been used in other studies, ^31^^,^^32^ obtaining the number of events allows for the extraction of key circadian features of the feeding behavior, such as period and rhythmicity. Future versions could include these data points as selectable options, to adjust to the experimenters’ needs.

Although our app offers in-site statistical analysis, output files can be obtained from all the analysis, allowing the export of processed data to other statistical software such as GraphPad Prism or Matlab. This could be particularly useful if in-depth statistical analysis is required, a feat that CRUMB is unable to achieve.

Finally, CRUMB is open-access, and the code is freely available, which hopefully will promote the use of this tool as a backbone for other functions required for the community. Although CRUMB was initially conceived with circadian analysis of feeding in mind, the app can be expanded to include analysis of food choices, also available in the FLIC system.

## Data Availability

Example data used in this paper are available as part of the CRUMB app on GitHub ( CRUMB GitHub). Zenodo: ClockLabX/CRUMB: Version 1.0 (v1.0.0), https://doi.org/10.5281/zenodo.7738298. ^41^ Data are available under the terms of the Creative Commons Attribution 4.0 International License (CC-BY-04). Source code available from: https://github.com/ClockLabX/CRUMB.git Archived source code at time of publication: https://doi.org/10.5281/zenodo.7738298. ^41^ License: MIT license.
