# Supplementary material for: CRUMB: a shiny-based app to analyze rhythmic feeding in Drosophila using the FLIC system
Source: F1000Res. 2023 Jun 19;12:374. Originally published 2023 Apr 6. [Version 2] doi: 10.12688/f1000research.132587.2 (PMC10314183; doi:10.12688/f1000research.132587.2)
Supplement: Peer review history

# Peer review: Reviewer response for version 2

**DOI:** 10.5256/f1000research.151442.r179838

**Published:** 2023-06-30

**Authors:** Abhilash Lakshman

**Affiliations:** 1 The Advanced Science Research Center, The City University of New York, New York, NY, USA

This is an important and timely contribution to the field. I thank the authors for considering the reviewers' comments and addressing them. I have no further comments to make.

Are the conclusions about the tool and its performance adequately supported by the findings presented in the article?

Yes

Is the rationale for developing the new software tool clearly explained?

Yes

Is the description of the software tool technically sound?

Partly

Are sufficient details of the code, methods and analysis (if applicable) provided to allow replication of the software development and its use by others?

Partly

Is sufficient information provided to allow interpretation of the expected output datasets and any results generated using the tool?

Partly

Reviewer Expertise:

organismal biology, chronobiology, animal behavior, neuroscience, computational biology

I confirm that I have read this submission and believe that I have an appropriate level of expertise to confirm that it is of an acceptable scientific standard.

# Peer review: Reviewer response for version 1

**DOI:** 10.5256/f1000research.145518.r168915

**Published:** 2023-04-21

**Authors:** Daniel J. Cavanaugh

**Affiliations:** 1 Department of Biology, Loyola University Chicago, Chicago, Illinois, USA

This article by Hidalgo and Chiu discusses the development of CRUMB, a novel software tool for analysis of data obtained with the Fly Liquid-Food Interaction Counter (FLIC) system. FLIC was developed for real-time monitoring of *Drosophila* interactions with a liquid food source. These interactions have previously been demonstrated to be highly correlated with feeding events, which allows for an automated assessment of feeding behavior over extended periods of time. The FLIC system therefore provides the opportunity to uncover the genetic and cellular underpinnings of circadian control of feeding behavior. This is important as it is becoming increasingly clear that circadian rhythms of feeding and metabolism have tremendous impact on overall organismal health and wellbeing.

 A current limitation of the FLIC system is the absence of user-friendly analysis tools. Existing analysis tools for the FLIC system require knowledge of the R programming language. Hidalgo and Chiu have therefore created CRUMB, a Shiny app that provides an easy-to-use Graphical user interface that performs standard FLIC analysis to identify feeding events and apply statistical methods to determine whether the data exhibit circadian patterns. This program will be extremely useful to the growing community of researchers using the FLIC system to monitor for circadian feeding patterns. CRUMB does an excellent job of producing intuitive data output files and graphs for data visualization. It also provides a streamlined pipeline that should enable speedier data processing compared with existing FLIC analysis programs. CRUMB could also serve to standardize data analysis, which will facilitate comparison of results across labs. A few small changes, detailed below, could make CRUMB even more useful:

- Data are extracted as feeding ‘events’ occurring within a user-defined bin length. As defined, events can be quite variable in length, potentially obscuring differences in total feeding time across the day. I recommend extracting ‘lick’ data in addition to events, and allowing the option to use binned ‘lick’ data in subsequent circadian analysis, as this will better capture dynamic changes in overall feeding throughout the day.
- One argument for the use of CRUMB is to standardize analysis across labs. It should be noted that the parameters shown in Figure 2A under “Select threshold for feeding events” are extremely important for accurate identification of feeding events. We found that parameter settings had to be determined empirically for our setup—for example, these differed significantly when moving from the original FLIC-MCU-1 to the FLIC-MCU-2. Although this would entail significant additional work on the authors’ part, it would be beneficial to build in an analysis that allows users to compare raw data to identified feeding events, to make sure that most apparent events are being captured with the parameter settings.
- Currently, statistical analysis of circadian rhythmicity is limited to autocorrelation testing. To allow for more direct comparison with locomotor activity data, it would be helpful to also offer chi-square periodogram, since the vast majority of papers reporting locomotor activity rhythms in *Drosophila *use this test. This would be beneficial but not essential, as the authors already note that data output files are available as CSV files, which can then be used for circadian analysis with other programs that offer more extensive circadian statistics.

Are the conclusions about the tool and its performance adequately supported by the findings presented in the article?

Yes

Is the rationale for developing the new software tool clearly explained?

Yes

Is the description of the software tool technically sound?

Yes

Are sufficient details of the code, methods and analysis (if applicable) provided to allow replication of the software development and its use by others?

Yes

Is sufficient information provided to allow interpretation of the expected output datasets and any results generated using the tool?

Yes

Reviewer Expertise:

Circadian neurobiology

I confirm that I have read this submission and believe that I have an appropriate level of expertise to confirm that it is of an acceptable scientific standard.

# Peer review: Reviewer response for version 1

**DOI:** 10.5256/f1000research.145518.r168914

**Published:** 2023-04-21

**Authors:** Abhilash Lakshman

**Affiliations:** 1 The Advanced Science Research Center, The City University of New York, New York, NY, USA

This manuscript by Hidalgo and Chiu is an important and timely contribution to the *Drosophila* rhythms community and describes a very useful and easy to implement application to analyze feeding rhythms in flies.  The application allows users to analyze data from the FLIC system and from what I understand the FLIC assay from Scott Pletcher’s lab is becoming a more popular tool to assay long-term feeding in flies, and therefore, this application will be an asset to the field.  I feel like the strengths of the application lay in, (i) the availability of several options for analyses, downloads, (ii) the ability to analyze multiple conditions simultaneously, (iii) the use of plotly which makes it easy for dynamic visualization of data, and (iv) that it is a freely available tool.

 Here are my specific comments that I feel may improve the manuscript:

- In the Introduction, the authors talk about several software options to analyze *Drosophila* locomotor activity rhythms, but do not cite several of the new and some old programs too.  It is important to have them cited here.
- Many of the packages used by the authors such as DT, plotly and shiny (and others) are lacking appropriate citations, which must be provided.
- Baseline data and QC section:  If I understand correctly, due to some constraints imposed by the detection system, some baselining is required.  The authors talk about two ways of baselining the raw data, i.e., running median and asymmetric least squares.  It is important for the authors to describe how these two are different and under what circumstances one should choose one or the other method.  For this manuscript to be a standalone resource for analyzing FLIC data, such clarification is critical.  Also, in my opinion while the binning interval is intuitive and obvious, it may not be particularly clear as to when using ‘mean’ binning function would be biologically meaningful/relevant.  The authors are requested to clarify these and provide examples of when one choice would make more sense than the other.
- In the parameter selection section, the authors talk about how the minimum threshold, feeding threshold, number of consecutive licks and gaps must be tailored to each monitor and need to be determined empirically.  This is not clear to me.  Based on what should one determine what is an acceptable parameter value?  Are there example citations?  The authors should cite and/or describe the influence of these settings on outcomes of the analyses and how this could affect downstream interpretations.  Maybe some examples would help.  For a reader coming across this paper, that would be crucial information.
- In the third section (Figure 3), there is an additional tab for binning length and binning function.  I don’t see any description of how this is different from the same input in section 1.  For example, can the binning length and function be different in sections 1 and 3?  Either way, it is important for the authors to state it explicitly.
- Also, the daytime and nighttime feature is great.  But how is the computation carried out for subjective day and nighttime under constant conditions if the users have provided long-term DD recording?  If this is not possible at the moment, a cautionary note would be important.
- In the ‘Use Case’ section, I am assuming that the autocorrelation period analysis is being performed for the four days in DD.  This is not clearly described in the text.  If this is indeed the case, the authors must clarify, while adding a disclaimer that four days is anyway too short for using time-series methods robustly.  If the authors have long-term (7-day) recording and they can use it, that would be great.  But it’s absolutely fine if they don’t have that.
- Also, in panels C and D, is the data considered only from LD?  If so, there is some other interesting thing going on here: if this is in LD, and there is no difference between day and nighttime feeding events in the clock mutant; does that mean that feeding rhythms, unlike locomotor activity, does not mask to light/dark transitions?  I think this may be worth pointing out because this adds a new dimension to the behavior that you have been able to identify using your new tool.  Also, I think showing the time-course of feeding activity over the entire duration of seven days may be useful to the reader.
- There also seems to be some discrepancy between the text and figure legend.  The text says three days in LD and four days in DD, and the legend says two days in LD and four days in DD.  Please clarify.

Are the conclusions about the tool and its performance adequately supported by the findings presented in the article?

Yes

Is the rationale for developing the new software tool clearly explained?

Yes

Is the description of the software tool technically sound?

Partly

Are sufficient details of the code, methods and analysis (if applicable) provided to allow replication of the software development and its use by others?

Partly

Is sufficient information provided to allow interpretation of the expected output datasets and any results generated using the tool?

Partly

Reviewer Expertise:

organismal biology, chronobiology, animal behavior, neuroscience, computational biology

I confirm that I have read this submission and believe that I have an appropriate level of expertise to confirm that it is of an acceptable scientific standard, however I have significant reservations, as outlined above.
